# On Remittent Ophthalmia—Strumous Ophthalmia of Children

**Published:** 1865-03

**Authors:** Henry Hancock

**Affiliations:** Surgeon to the Charing-Cross and Westminster Ophthalmic Hospitals, &c.


### Selections.

---

### ON REMITTENT OPHTHALMIA—STRUMOUS OPHTHALMIA OF CHILDREN.

---

By HENRY HANCOCK, Esq., Surgeon to the Charing-Cross and Westminster Ophthalmic Hospitals, &c.

---

[Many years ago, Mr. Hancock published some papers to show that what is commonly known as strumous ophthalmia does not depend upon that particular condition of constitution.]

The disease described under the titles "strumous," "scrofulous" or phlyctenular ophthalmia, is an affection of simple character, depending upon disorder of the digestive organs, influenced by the age of the patient, and presenting a type peculiar to diseases of the earlier period of life. True strumous or scrofulous ophthalmia is, as pointed out by Jacob, an affection of an entirely different character Phlyctenular ophthalmia attacks children of all conditions, whether scrofulous or otherwise; it does not appear to be influenced by station in life, nor is it especially restricted to any particular color of the hair or complexion; it attacks the fat, stout child, equally with the thin, and the highly-fed equally with the child of penury and want. In all cases the principal diagnostic signs of remittent fever are present; more urgent and well-marked, it is true, in some instances than in others, but in all they may more or less be traced, if ordinary attention be paid to the subject. The remissions of severity; increase of fever and heat towards night; the subsidence thereof, and accession of perspiration; the eruption about the nose and corners of the mouth; picking the nose and lips; rubbing the eyes; fœtid, sour, and disagreeable breath; the strawberry tongue, covered with moist fur; the enlarged liver; the tumid, hard, and swollen belly; irregularity of the bowels, sometimes constipated, at others relaxed; the evacuations clay-colored, or dark, or slimy, and mostly offensive. The tendency to eruptions over the face, head, and body, so invariably present in this form of ophthalmia, bears such strong affinity to the symptoms of the remittent fever of children, that I

have long regarded the ophthalmia of children as a disease of the same character, and have consequently discarded the term "strumous," and "scrofulous," as applied to it, designating it as *remittent* ophthalmia.

The term "strumous ophthalmia," has heretofore embraced a large class of cases. Mere congestion of the palpebral, or of the ocular conjunctiva, with or without phlyctenular or pustular eruption; inflammation of the sclerotica or of the cornea; ulceration; suppuration, or sloughing; interstitial deposit between the layers of the cornea; certain affections of the iris; and in some rare instances, of the retina, if accompanied by intolerance of light, having been classed under this designation. I propose, however, in this and subsequent papers, to consider first, the more simple form, the remittent ophthalmia; secondly, the "phlyctenular, or pustular ophthalmia," and lastly, the "strumous."

Remittent ophthalmia is the most frequent and simple, depending upon derangement of the digestive organs; it may attack any child in whom such derangement obtains. It is for the most part confined to the superficial tunics of the eye, and is very frequently accompanied by cutaneous eruptions. It may exist for a great length of time, without producing any serious permanent results. The complaint will often be confined to the conjunctiva of the eyelids, the edges of which are loaded with a viscid secretion. It its more severe form, the disease extends to the ocular conjunctiva, the redness being restricted to one or more fasciculi of superficial vessels, extending from the back of the eyeball to the margin or front of the cornea, where they commonly terminate in phlyctenulæ, which are either absorbed, leaving superficial white specks when seated in front of the cornea; or, suppurating and bursting, result in superficial or deep-seated ulceration, occasionally penetrating the entire thickness of the cornea, allowing the iris to protrude; or, in other cases, destroying a larger portion of the cornea, and changing its brightness altogether into a yellowish slough. These two latter forms are always succeeded by permanent adhesion of the iris to the cornea, and the formation of a cicatrix, which rarely disappears. The local symptoms in the milder forms, are pain; intolerance of light; redness; and the formation of simple pustules, with increased flow of tears. In the severer forms, ulceration, or even sloughing of the cornea will sometimes occur. In the majority of cases there is more of discomfort than of actual pain, although in some instances the pain is very severe. It is usually remittent, being absent during the day, recurring at night, and subsiding towards morning. In the early stages the pain is always sharp, darting, and intermitting; but when phlyctenulæ, pustules, or ulceration of the cornea exist, a continuous sensation of grit or sand in the eye obtains. The intolerance of light is a symptom so prominent and distressing, that many authors have been led to regard it as the cause, rather than the result of the disease. Experience, however, proves the error of this opinion; it is very true that with the subsidence of the intolerance of light a very marked improvement in the disease takes place; this, however, is not due to the subsidence of the intolerance of light, but to the removal of the cause which produced both it and the disease, the intolerance of light being merely a symptom of the disease, a sympathetic or functional affection, a morbid sensibility, depending upon disorder of the alimentary canal, and various secretions.

Although permanent and persistent in some, the photophobia is generally remittent; it appears to alternate with the pain, for whereas the latter is less urgent in the morning, and during the day, the intolerance of light is most severe at those times, subsiding towards evening, when the pain becomes more aggravated. The intensity of the photophobia, however, must not be regarded as any criterion of the severity of the disease; the less urgent and serious the malady, the greater, in most instances, will be intolerance of light. A child may open its eyes when considerable disorganization has taken place, but when the attack is comparatively slight, it will make great effort to exclude the minutest ray of light.

*Treatment.*—In the *British Medical Journal*, June 4, 1864, Mr. Cheshire, Senior Surgeon to the Birmingham and Midland Eye Hospital, in advocating the use of tartarised antimony as a remedy in strumous ophthalmia, observes, "I am aware it has long been the practice of ophthalmologists to administer a single dose of tartarised antimony, as a beginning to the treatment of strumous ophthalmia, &c., &c., but it does not appear to have been resorted to as a remedy, *per se*, for the cure of strumous affections of the eye."

In the same Journal, for the 25th June, 1864, Mr. Price, of Margate, in eulogising Mr. Cheshire's notice, adds, "That the plan of treatment therein recommended, is both little known and little practised, I am fully convinced; and my object in again bringing the subject forward is to endorse the statements of Mr. Cheshire, in the hope of inducing practitioners to follow this mode of treatment on a more extended scale."

It is very evidentthat Mr. Cheshire and Mr. Price had either forgotten, or had not read my lectures published in the *Lancet*, in the year 1852, in which I gave the result of my treatment during several previous years of strumous ophthalmia (so-called), by tartarised antimony, in opposition to the then existing treatment of that disease by tonics, and the local application of arg. nitratis, &c. Since the year 1849, I have not only steadily pursued this treatment, but strenuously recommended its adoption by the numerous surgeons who, from time to time visit the Royal Westminster Ophthalmic Hospital. Under these circumstances, I may be excused for again bringing forward the substance of the treatment I have so many years advocated.

The main objects to be steadily borne in mind in the treatment of remittent (strumous) opthalmia, are, relief of the organs of digestion, and the correction of the secretions. If these are attained, the other symptoms will improve, and the eyes share in the general change for the better; but unless a systematic plan, embracing diet, clothing, good nursing, as well as medicine, be adopted, little benefit will result. As I have before observed, remittent ophthalmia may, as a general rule, always be traced to a disordered condition of the digestive organs, a want of digestive power, an incapability of properly assimilating the food. The liver is sluggish, irregular in its action, and frequently enlarged; the mesenteric glands are commonly increased in size; the secretions are vitiated, and the blood consequently becoming impoverished and impure, the whole system is deranged. The child is rendered pale, weak, and fractious; its appetite is lost, or very capricious, and it is said to be strumous, weak, and delicate; but this condition need not become permanent, if the case be judiciously treated; if it is not at the outset erroneously supposed to depend upon cachexia or debility. The debility is for the most part more apparent than real, and procceds from irritation, or of oppression rather than of actual weakness. Remove the cause, and the debility will disappear *pari passu*.

I will not here enter upon the local treatment usually resorted to, as I have long ceased to employ it in remittent ophthalmia, excepting where the cornea has given way and the iris protrudes; in such cases, touching the protruding iris with the pointed nitrate of silver and applying the extract of belladona over the eyebrow are very useful. From repeated trals of the various local remedies recommended, I am convinced that they do more harm than good, causing great suffering without compensating benefit; indeed, children get well much more quickly when they are not used at all. The treatment shoud commence with an emetic of tartarised antimony, given as advised by Dr. Mackenzie, viz., in minute doses at frequent intervals, until free vomiting is excited; and this in all cases, however attenuated or however stout the child may appear, the object being to relieve the stomach of its offensive contents and to render its secretions more natural; to correct and restore the secretions of the kidneys, liver, and other glandular structures, and to influence the capillary system generally, so as to improve the condition of the skin and mucous membrane, and allay the morbid excitement of the nervous system.

This is best done by the tartarised antimony given as above, and it is very important that attention should be paid to the mode of its administration. The mere inducement of vomiting, the simple evacuation of the contents of the stomach is not sufficient, unless the medicine by being absorbed into the blood is enabled to produce other results; hence I have not observed anything like the same amount of good to result from sulphate of zinc, nor the ipecacuanha powder, or even from the tartarised antimony when the latter is administered in a single large dose to cause immediate vomiting. The following is the form which I usually employ:—

℞. Antim. tart. gr. iv.; syrup. ʒiv.; aq. cinnamoni ℥iij.; aq. distill. ad. ℥viii.

For a child under three years of age, two teaspoonfuls should be given every ten minutes until vomiting ensues; above this age, a tablespoonful should be given at similar intervals. It commonly requires four or five doses to produce the full effect, in some instances more, in a few less. This treatment should be repeated daily, until the intolerance of light begins to diminish, which it will mostly do after the first or second day, although in some of the more obstinate cases, four, or even six days will elapse before the desired result is obtained. When, however, the intolerance of light begins to subside, the emetic should be discontinued, and a powder of calomel and rhubarb, or mercury with chalk, and compound scammony powder every night, or every alternate night, until the tongue becomes clean, the abdomen softer and flatter, and the alvine secretions healthy; as these changes are brought about, the eyes will improve if attention be paid to the diet. When the attack is complicated with skin eruptions, aperients will be required for a somewhat longer period. Should the attack of ophthalmia be preceded by measles, small-pox, scarlet fever, or any other complaint of a highly depressing character, or if the child has been exposed to excessive want, is much attenuated, and covered with a cold clammy perspiration, calomel, even in small doses, should not be given, except with great caution; in no instance should more than one dose be given without the patient being seen, as should ptyalism take place, such extensive sloughing of the fauces, gums, and even of the lips and cheeks may ensue as to destroy the child in a very few days. I have known this occur more than once. With such children it is safer to abstain from the use of calomel altogether, and to substitute the compound scammony powder, with taraxacum and henbane. When mercury, however, is especially indicated, I always combine it with quinine, taking care not to push the medicine to such an extent as to produce ptyalism.—*Journal of British Ophthalmology, October* 1864, p. 35.

